# Parvovirus B19 Infection and Severe Anemia in Renal Transplant Recipients

**DOI:** 10.1100/2012/102829

**Published:** 2012-04-24

**Authors:** Antonio Carraturo, Valentina Catalani, Donatella Ottaviani, Patrizia Menichelli, Maurizio Rossini, Delia Terella, Brunello Biondi

**Affiliations:** ^1^Clinical Pathology Laboratory, Santa Maria Goretti Hospital, AUSL Latina, 04100 Latina, Italy; ^2^CEREM, Experimental Zooprophylactic Institute of Umbria and Marche (IZSUM), 60126 Ancona, Italy; ^3^Kidney Transplant Unit, Santa Maria Goretti Hospital, AUSL Latina, 04100 Latina, Italy

## Abstract

Kidney transplant (KT) recipients can develop symptomatic Parvovirus (PV) B19 infections, frequently associated with persistent anemia. The aim of this study was to evaluate the prevalence and clinical significance of PV B19 infection in anemic and non-anemic KT patients. Overall, out of 64 patients monitored for the presence of PV B19 by real-time PCR, 2 (3.12%) had an active PV B19 infection, in absence of other viral coinfections. The 2 cases occurred in nonanemic kidney transplant patients group (2/50, 4%), while none of the anemic transplant patients (0/14) was found to suffer from this infection. Moreover, patients affected by active PV B19 infection showed viral loads not exceeding 1 × 10^5^ genome copies/reaction. In conclusion, in this study, PV B19 infection was not common in renal transplant population and wasn't associated with severe anemia.

## 1. Introduction

Anemia is a frequent problem after renal transplantation: up to 39% of kidney transplant (KT) recipients suffer from chronic anemia and, of these patients, 9% suffer from a severe form, characterized by hemoglobin levels ≤11 g/dL for males and ≤10 g/dL for females [1, 2]. Many evidence suggests that the anemic state in transplant recipients can also be caused by Parvovirus (PV) B19 infection [3, 4]. Discovered in 1975, PV B19 is a small, nonenveloped, single-stranded DNA virus belonging to the *Parvoviridae* family [5]. This is a common pathogen in humans, and the expression of the infection depends on the host's hematological and immunologic status. In immunocompetent children, PV B19 is the etiologic agent of erythema infectiosum (fifth disease). In healthy pregnant women it causes hydrops fetalis. In immunosuppressed patients, including organ transplant recipients, B19 virus can persist for years due to impairment of the neutralizing antibody response and/or cellular immunity and it may be associated with chronic clinical manifestations, such as anemia and other cytopenias [3, 4].

In particular, KT recipients may acquire symptomatic PV B19 infection from the donor, from the community, or from reactivation of endogenous latent or persistent virus [6]. Although numerous cases of PV B19 infection in renal transplant patients have been reported [2, 7], the clinical burden of PV B19 infection is not well characterized. Moreover, the association between PV B19 infection and anemia in KT recipients remains to be clarified [2].

To address these issues, we evaluated the prevalence and clinical significance of Parvovirus B19 infection in anemic and nonanemic patients who had received a renal transplant for at least 6 months. We chose these patients because most published studies have assessed the occurrence of PV B19 infection in KT recipients within a 6-month period after transplantation, when immunosuppression is stronger, while only few studies have been performed in patients belonging to the population that we selected.

## 2. Methods

From January to July 2008, 128 blood samples from 64 informed KT patients attending to Santa Maria Goretti Hospital in Latina, Italy, were collected. Of these patients (39 males, 25 females, aged 25–67), who had received a kidney transplant for at least 6 months, 14 suffered from unexplained severe anemia, with hemoglobin levels ≤11 g/dL in males and ≤10 g/dL in females. Two blood samples for each patient were taken (the second 3 months after the first). All the samples were analyzed for the presence of PV B19 DNA by quantitative real-time PCR. Viral DNA was extracted from 200 *μ*L EDTA-anticoagulated whole blood with the COBAS AmpliPrep instrument using the TNAI (Total Nucleic Acids Isolation) Kit (both Roche Diagnostics GmbH, Mannheim, Germany). The amount of Internal Control (IC) was 3.1 *μ*L per 50 *μ*L of quantitation standard (QS) diluent [8]. The eluates (5 *μ*L) containing viral nucleic acids were analyzed by real-time PCR on the LightCycler instrument using the LightCycler Parvovirus B19 Quantification Kit (Roche Diagnostics), according to the manufacturer's instructions. Samples positive for PV B19 DNA were also tested to detect the possible presence of concomitant active viral infections. Detection and quantification by real-time PCR method of hepatitis C virus (HCV), hepatitis B virus (HBV), and human immunodeficiency virus (HIV), were performed using the fully automated system COBAS Ampliprep/COBAS TaqMan (Roche Diagnostics). For Cytomegalovirus (CMV) detection, the COBAS Amplicor CMV Monitor Test was used (Roche Diagnostics), with nucleic acid extraction performed on the Ampliprep instrument using the TNAI kit (Roche Diagnostics). For Polyomavirus JC/BK, Epstein-Barr virus (EBV), and Herpes simplex virus type 1 and type 2 (HSV) detection and quantification, viral DNA was extracted by the automated MagNA Pure LC system (Roche Diagnostics) according to the manufacturer's instructions and the real-time PCR reactions were performed on the LightCycler instrument using LightMix Kit Polyomaviruses JC and BK (TIB MOLBIOL, Berlin, Germany), LightCycler EBV Quantification Kit (Roche Diagnostics), and LightCycler HSV 1/2 Detection Kit (Roche Diagnostics), respectively. Statistical analysis was performed using Fisher exact test, with *P* ≤ 0.05 required for significance.

## 3. Results and Discussion

The results are summarized in Table 1. Overall, out of 64 KT recipients, 2 (3.12%) were affected by active Parvovirus B19 infection, both belonged to the nonanemic patients group. In particular, the prevalence of PV B19 infection was 4% (2/50) in nonanemic patients compared to 0% (0/14) in anemic patients. This result is not supported by statistical significance (Fisher exact test *P* = 0.6), probably because of the small sample size.

The first patient affected by active PV B19 infection was a 60-years-old man on dialysis treatment since 1992 who received a deceased donor kidney in 1998. He was using cyclosporine and mycophenolate-mofetil as immunosuppressive drugs and his value of serum creatinine was 1.3 mg/dL. During PV infection his hematocrit was 37% from a baseline of 44% with an hemoglobin value decreased from 14 to 12.4 g/dL and PV B19 viremia of 1 × 10^5^ genome copies/reaction. Before the diagnosis of Parvovirus B19 infection, he suffered from myalgia, abdominal pains, arthralgias, and recurrent fevers. After treatment with Immunoglobulin (IVIG), viremia fell below detection limit and his hematocrit and hemoglobin levels returned to normal.

The second patient was a 62-years-old man who received a deceased donor kidney in 2001, after 4 years of dialysis. He was receiving sirolimus and mycophenolate-mofetil as immunosuppressive treatment and his serum creatinine was 2.30 mg/dL. This patient showed no clinical signs or hematological disorders having hemoglobin and hematocrit values of 14 g/dL and 41%, respectively. His PV B19 viremic titer was 1 × 10^4^ genome copies/reaction. After reduction of immunosuppressive medication, viremia fell to undetectable levels and the patient recovered.

In these two patients affected by active PV B19 infection, Polyomavirus JC/BK, CMV, EBV, HSV, HCV, HBV, and HIV, were not detected.

Previous studies have shown that the prevalence of PV B19 infection in KT patients ranged from 0 to 6.3% [9–11], while reached 23% in anemic KT patients [12]. In agreement with these findings, we found 2 positive cases out of 50 nonanemic KT patients (4%). It is to be noted that nonanemic patients positive for PV B19 investigated here showed viral loads not exceeding 1 × 10^5^ genome copies/reaction. Our results are consistent with a previous study that correlated only the viremic titers higher than  1 × 10^6^  copies/reaction with severe anemia [13]. Surprisingly, in our study, none (0/14) of KT patients with severe anemia had an active infection by Parvovirus B19, although one PV B19 positive patient showed a moderate reduction in hemoglobin concentration and in hematocrit. However, it is to underline that it is difficult to compare the results of different studies because of the heterogeneity in definition of posttransplant anemia [6].

In conclusion, we can support that results of the present study are partially consistent with data reported in the literature, although Parvovirus B19 infection was not common in the renal transplant population and wasn't associated with severe anemia. We believe that could be important routinely performing a differential diagnosis for Parvovirus B19 in all cases of posttransplant anemia and/or heavy immunosuppression regimen.

## Figures and Tables

**Table 1:** Prevalence of Parvovirus B19 in kidney transplant recipients, from January to July 2008, Latina, Italy.

	Patients			
	Nonanemic		Anemic	
No. of patients	50		14	
	Male 31	Female 19	Male 8	Female 6
No. of PV B19 positive patients	2	0	0	0
